# Radiomic features of peri-left atrial epicardial adipose tissue and atrial fibrillation recurrence after ablation

**DOI:** 10.1136/openhrt-2025-003364

**Published:** 2025-07-08

**Authors:** Yifan Hu, Longzhe Gao, Qiangrong Wang, Jin Chen, Shanshan Jiang, Genqing Zhou, Jiayin Zhang

**Affiliations:** 1 Dongtai People’s Hospital, Yancheng, Jiangsu, China; 2 Cardiology, Shanghai General Hospital, Shanghai, China; 3 Bayer HealthCare Co Ltd, Beijing, China; 4 Radiology, Shanghai General Hospital, Shanghai, China

**Keywords:** Atrial Fibrillation, Computed Tomography Angiography, Biomarkers

## Abstract

**Abstract:**

**Objectives:**

This study aimed to establish a prediction model that incorporates the radiomic features of epicardial adipose tissue (EAT) to predict atrial fibrillation (AF) recurrence after ablation.

**Methods:**

We prospectively enrolled patients with AF who underwent pulmonary CT venography before ablation therapy at two hospitals (470 patients in the internal cohort and 81 in the external cohort) between June 2018 and December 2019. Stepwise regression was used to identify clinically relevant factors, including quantitative EAT and left atrial (LA)-EAT measurements (model 1). The random forest algorithm was used to select the radiomic features of EAT and LA-EAT. A radiomics model predicting AF recurrence within 1 year after ablation was developed using these features (model 2). Subsequently, logistic regression was used to integrate radiomic features with clinical data (model 3).

**Results:**

In total, 551 patients were enrolled (median age: 66 years, IQR: 60–72 years; 340 men), with 145 experiencing AF recurrence within 1 year. Model 2, based on LA-EAT radiomic features, demonstrated significantly better performance than model 1 (clinical predictive factors and LA-EAT volume) for predicting AF recurrence (areas under the curve (AUC): 0.737 vs 0.584 in the external validation cohort). Model 3 exhibited the highest performance (AUC=0.790 in the external validation cohort, sensitivity value=0.800). Additionally, the combined model provided the highest net clinical benefit within a threshold probability range of 0.2–0.4.

**Conclusions:**

The LA-EAT radiomics model along with LA-EAT volume and clinical risk factors exhibited the highest predictive performance for AF recurrence following ablation therapy.

**WHAT IS ALREADY KNOWN ON THIS TOPIC**

- Previous studies have associated clinical factors and quantitative epicardial adipose tissue (EAT) measurements with atrial fibrillation (AF) recurrence after ablation, but predictive models combining these parameters lack robustness and accuracy.

**WHAT THIS STUDY ADDS**

- This study identifies peri-left atrial EAT (LA-EAT) radiomic features from pulmonary CT venography (PTCV) as optimal predictors of AF recurrence. A combined model integrating these radiomic features, clinical factors (age, elevated B-type natriuretic peptide (BNP)/pro-BNP) and LA-EAT volume achieved the highest predictive accuracy (external validation area under the curve=0.790).

**HOW THIS STUDY MIGHT AFFECT RESEARCH, PRACTICE OR POLICY**

- This study could transform clinical research, practice and policy by enabling more precise risk stratification. Integrating radiomics into AF ablation planning allows for the early identification of high-risk patients, which in turn promotes proactive monitoring—such as using implantable cardiac monitors—to detect asymptomatic AF recurrence. This early detection can lead to timely interventions that preserve cardiac function and inform updates to postablation surveillance protocols.

## Introduction

 The incidence of atrial fibrillation (AF), the most common arrhythmia, has steadily increased, and AF is strongly associated with severe complications, such as stroke and heart failure. Additionally, it imposes a substantial public health burden.^1 2^ Catheter ablation is the cornerstone of AF treatment,^3^ significantly reducing adverse clinical events and enhancing patients’ quality of life.^4^ Nevertheless, there is a possibility of recurrence following ablation.

Persistent AF significantly increases the risk of ischaemic stroke or systemic embolism, and asymptomatic subclinical AF, which is more common after catheter ablation, is no exception.^5^ Traditional short-term monitoring methods are insufficient to accurately detect AF recurrence, often leading to overestimation of ablation success rates.^6^ Additionally, prediction models based on 10 scoring systems incorporating clinical factors, such as age, sex, hypertension, diabetes and systemic inflammation, have demonstrated only moderate predictive performance, with areas under the curve (AUC) ranging from 0.553 to 0.669.^7^

In addition to conventional clinical factors, recent studies have highlighted the significant role of left atrial (LA) and epicardial adipose tissue (EAT) characteristics, including thickness, volume and density, in AF occurrence and postablation recurrence.^8 9^ EAT, a metabolically active fat depot, may influence atrial remodelling through mechanical compression, paracrine signalling, and the release of inflammatory mediators.^10^ The quantitative evaluation of EAT offers new insights into studies on AF,^11^ particularly in relation to the peri-LA EAT (LA-EAT), which is closely associated with the pulmonary artery.

Recent studies have identified increased periatrial EAT, specifically LA-EAT, as the strongest predictor of AF recurrence.^12^ Furthermore, radiomics-based quantitative assessment of LA-EAT density has shown superior predictive performance for postablation AF recurrence compared with overall EAT metrics.^13^ These findings underscore the unique prognostic value of LA-EAT in assessing AF recurrence risk. However, a comprehensive predictive model integrating LA-EAT radiomics features with traditional clinical factors to accurately assess AF recurrence risk has not been developed.

To address this limitation, our study used an automated segmentation approach based on deep learning to investigate the relationship between radiomics features of LA-EAT and overall EAT with AF recurrence following radiofrequency ablation (RFA). Additionally, we aimed to develop predictive models for AF recurrence based on LA-EAT and EAT characteristics to improve the management of patients with AF and support personalised treatment strategies.

## Methods and materials

### Study population

Patients who underwent a first-time RFA for AF were recruited from hospital A between June 2018 and December 2019 and from hospital B between January 2021 and December 2022.

**Figure 1:**
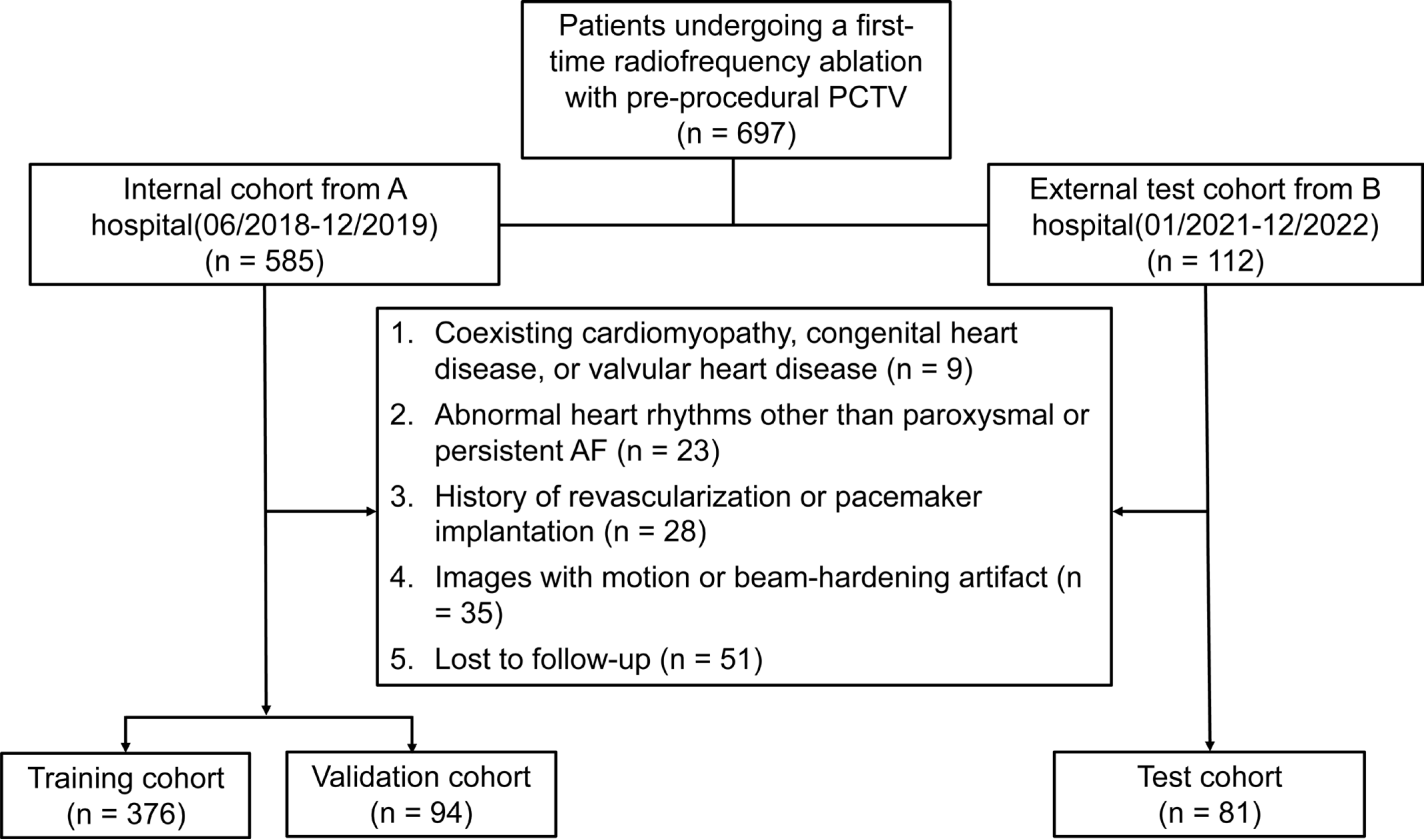
Inclusion and exclusion criteria for the internal and external cohorts. AF, atrial fibrillation; PCTV, pulmonary CT venography.

The inclusion criteria for this study were (1) adult patients (≥18 years old); (2) successful AF ablation, defined as complete electrical isolation of the pulmonary veins from the left atrium, with intraoperative confirmation of bidirectional conduction block at all targeted sites and (3) the availability of pulmonary CT venography (PCTV) imaging data obtained within 1 week before the procedure, along with complete medical records. The exclusion criteria were (1) arrhythmias other than paroxysmal AF (intermittent AF terminating within ≤7 days of onset) or persistent AF (continuous AF sustained for >7 days, requiring intervention), as defined by the 2023 ACC/AHA/ACCP/HRS Guideline (ACC = American College of Cardiology; AHA = American Heart Association; ACCP = American College of Chest Physicians; HRS = Heart Rhythm Society) for the Diagnosis and Management of AF (14); (2) loss to follow-up; (3) severely impaired PCTV image quality; (4) coexisting cardiomyopathy, congenital heart disease or valvular heart disease and (5) history of coronary revascularisation or pacemaker implantation. A detailed flow chart of the patient selection process is shown in figure 1.

### CT acquisition protocol

For the internal cohort, all patients were scanned using a 256-slice wide detector CT scanner (Revolution HD, GE Healthcare, USA) in nongated helical mode, covering from the thoracic inlet to the diaphragm. A 50–60 mL contrast media bolus (Iomeprol, Iomeron, 400 mg iodine/mL, Bracco, Italy) was injected into the antecubital vein at a rate of 4–5 mL/s, followed by flushing with 40 mL of saline. The detailed scan parameters were as follows: collimation=256×0.625 mm, reconstructed slice thickness=0.625 mm, reconstructed slice interval=0.5 mm, rotation time=280 ms and matrix=512×512 pixels. In addition, we applied automated tube voltage and current modulation (KV Assist, Smart mA, GE Healthcare, USA).

For the external test cohort, PCTV data were acquired on a second-generation dual-source CT scanner (SOMATOM Definition Flash, Siemens Healthineers, Germany) via a nongated helical mode. We employed contrast injection and triggering techniques similar to those used in the internal training cohort. The detailed scan parameters include collimation=64×0.6 mm, reconstructed slice thickness=0.75 mm, reconstructed slice interval=0.5 mm and rotation time=280 ms. Moreover, we applied automated tube voltage and current modulation (CAREKv, CAREDose 4D; Siemens Healthineers, Germany).

### Radiomics feature extraction

PCTV images were processed using an automated nnU-Net-based segmentation model. From the initial EAT segmentation results,^14^ 96 manually annotated LA-EAT cases were extracted to train a secondary nnU-Net-based segmentation model (online supplemental appendix table S1). This secondary model was subsequently applied to all images to ensure consistent LA-EAT segmentation results.

Radiomic features were extracted from PCTV images using the PyRadiomics package in Python (V.3.9.12). Feature definitions and extraction parameters followed PyRadiomics documentation (https://pyradiomics.readthedocs.io/en/latest/) and are detailed in online supplemental appendix table S2.

### Radiomics and clinical feature selection

**Figure 2:**
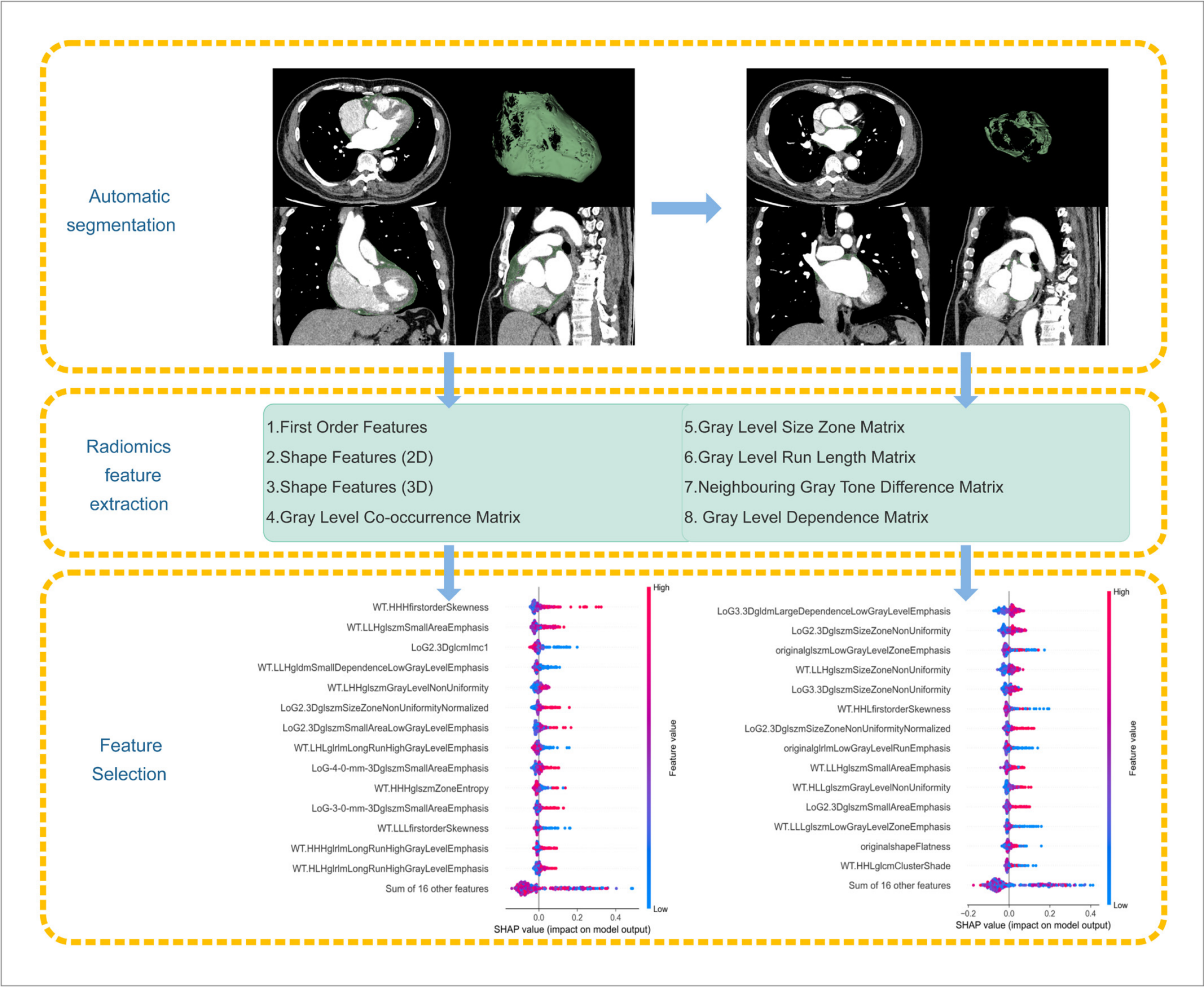
Flow chart of key steps in radiomics. The left side illustrates the schematic segmentation of the entire EAT, whereas the right side shows the segmentation of the LA-EAT. Both follow the same feature extraction and dimensionality reduction processes. The SHAP beeswarm plot shows the top 15 important features based on their average impact on the model predictions. Each point represents an individual instance, and colours indicate feature values (red for high and blue for low). The horizontal spread of the points indicates how feature variations affect the output of the model. EAT, epicardial adipose tissue; LA, left atrial; SHAP, shapley additive explanations.

To ensure consistency across different institutions, combat harmonisation was applied separately to the radiomic features of LA-EAT and total EAT. The internal dataset was then randomly divided into training and validation cohorts in an 8:2 ratio. Radiomic features in the training cohort were standardised, and the same parameters were subsequently used to standardize the validation and external test cohorts. Feature selection was performed using a random forest (RF) algorithm to rank features by importance scores. Following the retention of the top 30 features, mutual information criteria were applied to eliminate redundancy while preserving 80% of the cumulative information content. A summary of these key processes is shown in figure 2.

Apart from the radiomics features, LA-EAT/EAT quantification (including density and volume), demographic and clinical variables, including AF type, age, sex, body mass index (BMI), clinical history (eg, impaired cardiac function, coronary artery disease, hypertension, dyslipidaemia, diabetes), smoking history, alcohol consumption history, CHA₂DS₂-VASc score and laboratory parameters (B-type natriuretic peptide (BNP), pro-BNP, and C reactive protein levels), were collected and initially screened through univariate analysis for their association with AF recurrence. Stepwise regression was applied after evaluating multicollinearity (using the variance inflation factor), excluding variables with weaker univariate significance and selecting those with the lowest Akaike information criterion scores. Finally, well-established clinical factors known to be associated with AF recurrence were incorporated into the clinical model. See the online supplemental appendix for specific definitions.

### Model development and testing

Logistic regression (LR) models were used to develop model 1, which incorporated clinical factors and LA-EAT/EAT quantification. LR, RF and support vector machine (SVM) classifiers were used to construct radiomics models for predicting AF recurrence within 1 year. Hyperparameter tuning was performed using a grid search with fivefold cross-validation.

The best-performing radiomics model (model 2) in the validation cohort was integrated with the clinical model using a multivariate LR approach, with the predicted probabilities from both models serving as input features (model 3). Model performance was evaluated in the validation and external test cohorts using receiver characteristic curve analysis, with key metrics including AUC, sensitivity, specificity, accuracy and F1 score. Calibration curves were generated to evaluate prediction consistency, and decision curve analysis (DCA) was performed to assess clinical utility.

### Study endpoint and patient follow-up

Patients were followed up at 3, 6 and 12 months after the procedure. During each visit, arrhythmia-related symptoms were assessed, and the patients underwent 12-lead electrocardiography and 24-hour Holter monitoring. Patients who experienced AF-related symptoms between scheduled visits were recalled for further evaluation. The study endpoint was defined as AF recurrence within 12 months of a 3-month blanking period, including symptomatic or documented AF and other atrial arrhythmias.

### Statistical analysis

Statistical analyses of the clinical data were conducted using R software (V.4.2.1). Continuous variables are expressed as mean±SD for normally distributed data and as median with IQR for non-normally distributed data.

Continuous variables were compared between the training and validation cohorts using either the independent-sample t-test or the Mann-Whitney U test, depending on data distribution, which was assessed using the Shapiro-Wilk test. Categorical variables were compared using the χ^2^ test.

## Results

### Patient characteristics

A total of 697 patients from two medical institutions who met the inclusion criteria were initially reviewed. A total of 146 patients meeting the exclusion criteria were excluded (figure 1), resulting in a final cohort of 551 patients (median age: 66 years, IQR: 60–72 years; 340 males). Among them, 305 patients (55.4%) presented with paroxysmal AF, whereas 246 (44.6%) had persistent AF. During the 1-year follow-up, AF recurrence was observed in 145 patients. Significant differences between the internal and external cohorts were observed in terms of dyslipidaemia, smoking history, age, elevated BNP or pro-BNP levels and LA-EAT. Further details are presented in table 1.

**Table 1:** Baseline clinical characteristics and EAT quantitative data

Baseline clinical information	Internal cohort (n=470)	External cohort (n=81)	P value
AF type			
Paroxysmal AF	256 (54.5%)	49 (60.5%)	0.375
Persistent AF	214 (45.5%)	32 (39.5%)	
Recurrence	125 (26.6%)	20 (24.7%)	0.824
Age	66 (60, 72)	66 (60,72)	0.707
Gender			
Male	293 (62.3%)	47 (58.0%)	0.539
Female	177 (37.7%)	34 (42.0%)	
BMI	24.91 (23.03, 27.04)	25.70 (23.22, 27.60)	0.232
Clinical history			
Impaired cardiac function	84 (17.9%)	8 (9.8%)	0.105
Coronary heart disease	71 (15.1%)	8 (9.8%)	0.285
Hypertension	288 (61.2%)	26 (69.1%)	0.221
Dyslipidaemia	213 (45.3%)	48 (59.3%)	0.028
Diabetes	87 (18.5%)	14 (17.3%)	0.914
Smoking	95 (20.2%)	8 (9.8%)	0.040
Alcohol	74 (15.7%)	7 (8.6%)	0.134
CHA2DS2_VASc	2 (1, 3)	2 (1, 3)	0.679
Elevated BNP or pro-BNP	275 (58.5%)	59 (72.8%)	0.020
C reactive protein	1.30 (0.70, 2.60)	1.50 (0.80, 4.00)	0.217
LA-EAT quantification			
Density (HU)	−85.88 (−89.00, –82.15)	−84.28 (−87.63, –82.42)	0.126
Volume (cm^3^)	18.27 (13.64, 25.29)	16.39 (12.744, 22.15)	0.036
EAT quantification			
Density (HU)	−85.29 (−88.17, –82.44)	−86.37 (−90.95, –83.07)	0.064
Volume (cm^3^)	132.26 (103.93, 170.68)	132.13 (89.45, 166.99)	0.376

Age refers to the patient’s age at the time of radiofrequency ablation.

* The CHA₂DS₂-VASc score assesses stroke risk in atrial fibrillation patients, with points assigned for: Congestive heart failure (1), Hypertension (1), Age≥75 (2), Diabetes (1), Stroke/TIA (2), Vascular disease (1), Age 65-74 (1), and Female sex (1). Higher scores indicate greater stroke risk.

AF, atrial fibrillation; BMI, body mass index; BNP, B‐type natriuretic peptide; EAT, epicardial adipose tissue; LA, left atrial.

### Clinical factors selection

Univariate LR analysis revealed that BMI, EAT volume and LA-EAT volume were significantly associated with AF recurrence within 1 year after RFA, with LA-EAT showing a stronger association than EAT. In multivariate analysis adjusted through stepwise regression, age and LA-EAT volume were two independent predictors of AF recurrence within 1 year after RFA (table 2). Among these, age and LA-EAT volume were considered independent risk factors. Although other well-established predictors such as BMI, AF type and sex did not demonstrate significant associations with recurrence in this cohort, they were included in model 1 for their potential discriminatory power.

**Table 2:** Univariate and multivariable LR in internal cohort (recurrence within 3–12 months postablation)

Variable	No recurrence(n=345)	Recurrence(n=125)	Univariate LR			Multivariable LR		
			HR	95% CI	P value	HR	95% CI	P value
AF type (persistent AF)	147 (42.6%)	67 (53.6%)	1.46	0.93 to 2.31	0.099	–	–	–
Age at ablation	67(61, 73)	65(57, 71)	0.98	0.96 to 1.01	0.137	0.99	0.99 to 1.00	0.014
Gender (male)	216 (62.6%)	77 (61.6%)	0.99	0.62 to 1.59	0.980	–	–	–
BMI	24.83 (22.82, 26.78)	25.28 (23.62, 27.46)	1.08	1 to 1.16	0.040	–	–	–
Impaired cardiac function	61 (17.7%)	23 (18.4%)	0.99	0.55 to 1.78	0.980	–	–	–
Coronary heart disease	53 (15.4%)	18 (14.4%)	0.9	0.47 to 1.74	0.758	–	–	–
Hypertension	212 (61.4%)	76 (60.8%)	1.04	0.65 to 1.65	0.876	–	–	–
Dyslipidaemia	155 (44.9%)	58 (46.4%)	1.05	0.67 to 1.65	0.840	–	–	–
Diabetes	64 (18.6%)	23 (18.4%)	1.08	0.61 to 1.93	0.785	–	–	–
Smoking	71 (20.6%)	24 (19.2%)	0.77	0.43 to 1.39	0.387	–	–	–
Alcohol	54 (15.7%)	20 (16.0%)	0.94	0.51 to 1.76	0.851	–	–	–
CHA2DS2_VASc	2 (1, 3)	2 (1, 3)	0.94	0.81 to 1.08	0.392	–	–	–
Elevated BNP or pro-BNP	193 (55.9%)	82 (65.6%)	1.24	0.78 to 1.97	0.365	1.09	1.00 to 1.18	0.052
C reactive protein	1.30 (0.80, 2.50)	1.40 (0.60, 2060)	0.97	0.91 to 1.04	0.357	–	–	–
LA-EAT density (HU)	−86.21 (–89.20, 82.24)	−85.29 (–87.91, –81.97)	1.03	0.99 to 1.08	0.178	–	–	–
LA-EAT volume (cm^3^)	17.57 (13.50, 23.31)	20.38 (14.59, 28.97)	1.03	1 to 1.05	0.030	1.01	1.00 to 1.01	0.018
EAT density (HU)	−85.44 (–88.17, –82.46)	−84.88 (–88.16, –82.43)	1.01	0.98 to 1.05	0.552	–	–	–
EAT volume (cm^3^)	128.92 (101.71, 165.77)	138.20 (107.70, 187.60)	1	1 to 1.01	0.051	–	–	–

* The CHA₂DS₂-VASc score assesses stroke risk in atrial fibrillation patients, with points assigned for: Congestive heart failure (1), Hypertension (1), Age≥75 (2), Diabetes (1), Stroke/TIA (2), Vascular disease (1), Age 65-74 (1), and Female sex (1). Higher scores indicate greater stroke risk.

AF, atrial fibrillation; BMI, body mass index; BNP, B‐type natriuretic peptide; EAT, epicardial adipose tissue; LA, left atrial ; LR, logistic regression.

### Model 1/model 2 validation and model 3 factor selection

**Figure 3:**
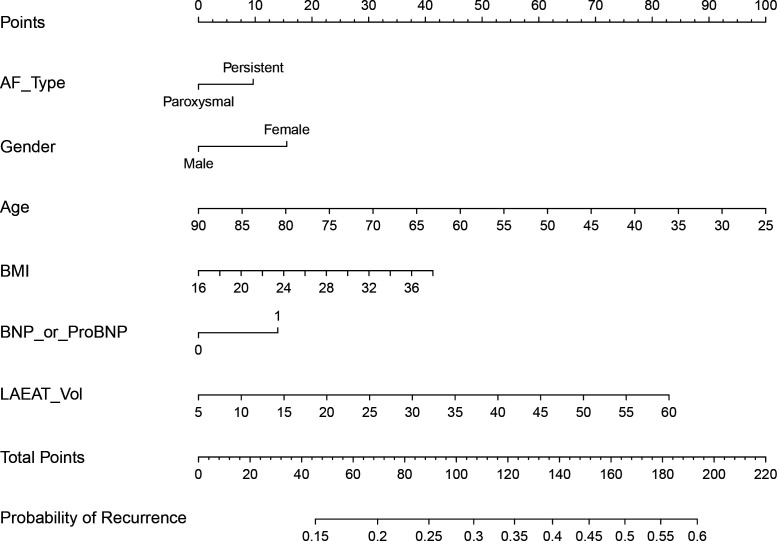
Clinical model nomogram for predicting recurrence within 1 year after RFA in patients with AF. The clinical nomogram presented here assigns a score to each selected predictor (including the LA-EAT volume) within the score range at the top of the plot. The total score is then matched with the scale at the bottom to estimate the probability of recurrence within 1 year after RFA in patients with AF. Age, LA-EAT volume and BNP/pro-BNP are the most significant factors identified through stepwise regression using the Akaike information criterion, whereas BMI, AF type and sex, recognised as influential factors, also predict AF recurrence. AF, atrial fibrillation; BMI, body mass index; BNP, B-type natriuretic peptide; EAT, epicardial adipose tissue; LA, left atrial; RFA, radiofrequency ablation.

Clinical predictive factors and LA-EAT volume were incorporated into an LR model (model 1). Model 1 demonstrated limited predictive performance, with accuracy rates of 0.564 and 0.580 and AUC values of 0.586 and 0.584 in the internal validation and external test cohorts, respectively. These results indicate that the clinical model alone was insufficient for accurately predicting AF recurrence. A nomogram was constructed to illustrate the contribution of individual risk factors to the prediction of AF recurrence (figure 3).

**Figure 4:**
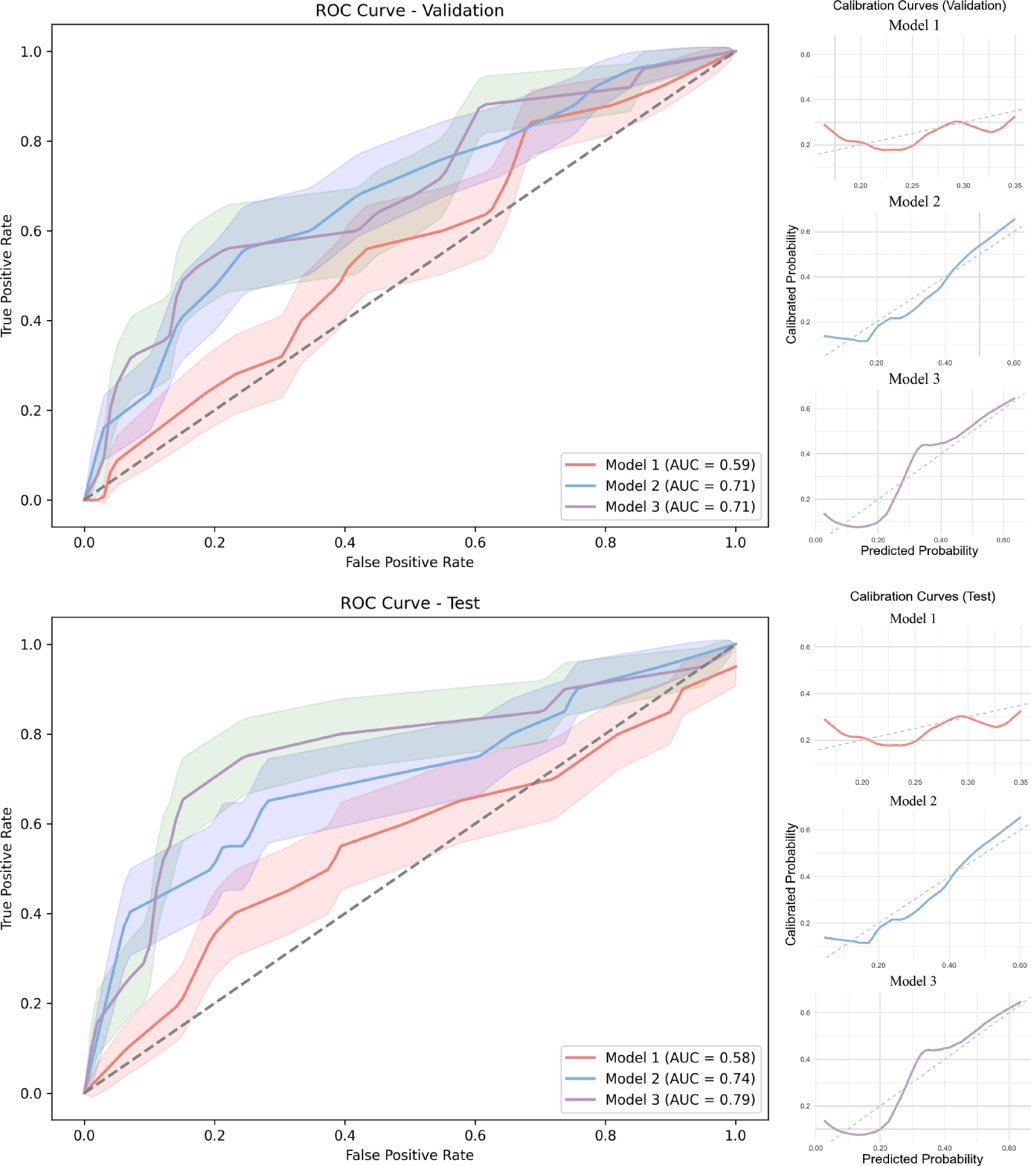
ROC and calibration curves for models 1, 2 and 3. Model 1 (clinical factors and LA-EAT volume), model 2 (radiomics model), model 3 (model 1+model 2). ROC curves showing the predictive performance of each model in internal validation and external test cohorts. Calibration curves depicting the agreement between predicted probabilities and actual outcomes for each model. AUC, area under the curve; EAT, epicardial adipose tissue; LA, left atrial; ROC, receiver operating characteristic.

In contrast, radiomics-based prediction models demonstrated significantly improved performance (figure 4). Feature selection identified 13 LA-EAT radiomics features and 16 EAT radiomics features. The LR model incorporating LA-EAT radiomics features achieved optimal sensitivity, with values of 0.760 (internal validation) and 0.750 (external test cohort), alongside AUCs of 0.713 and 0.737, respectively (table 3; detailed parameters in online supplemental appendix table S3). While the RF model exhibited near-perfect specificity (approaching 1.0), its limited sensitivity (~0.3) resulted in substantial underdetection of recurrence cases, restricting clinical applicability. The SVM model showed comparable overall performance to LR but with marginally reduced sensitivity. Consequently, the LA-EAT radiomics LR model (model 2) was selected for subsequent integration into the combined model 3.

**Table 3:** Diagnostic performance of clinical model, radiomics model and combined model

Metric	Clinic model	LA-EAT radiomics model			EAT radiomics model			Combined model
		LR	RF	SVM	LR	RF	SVM	Clinic+LR (LA-EAT)
Training								
Sensitivity	0.590	0.717	0.990	0.690	0.610	1	0.790	0.680
Specificity	0.594	0.601	1	0.616	0.627	1	0.851	0.663
Accuracy	0.593	0.630	0.997	0.636	0.622	1	0.835	0.668
F1-Score	0.435	0.505	0.995	0.502	0.462	1	0.718	0.521
AUC	0.631	0.717	1	0.718	0.673	1	0.888	0.727
Validation								
Sensitivity	0.560	0.760	0.240	0.720	0.640	0.320	0.480	0.680
Specificity	0.565	0.507	0.942	0.507	0.507	0.928	0.667	0.536
Accuracy	0.564	0.575	0.755	0.564	0.543	0.766	0.617	0.575
F1-Score	0.406	0.487	0.343	0.468	0.427	0.421	0.400	0.460
AUC	0.586	0.713	0.788	0.715	0.566	0.561	0.542	0.714
Testing								
Sensitivity	0.600	0.750	0.300	0.750	0.600	0.100	0.500	0.800
Specificity	0.574	0.623	0.951	0.639	0.574	0.934	0.672	0.689
Accuracy	0.580	0.654	0.790	0.667	0.580	0.728	0.630	0.716
F1-Score	0.414	0.517	0.414	0.526	0.414	0.154	0.400	0.582
AUC	0.584	0.737	0.795	0.737	0.596	0.663	0.632	0.790

AUC, area under the curve; EAT, epicardial adipose tissue; LA-EAT, left atrial ; LR, logistic regression; RF, random forest; SVM, support vector machine.

### Performance evaluation and model comparison

In the internal validation cohort, model 3 demonstrated significantly superior discriminative performance compared with model 1 (DeLong’s test: p=0.050). However, no statistically significant differences were observed between model 2 and either model 1 (p=0.129) or model 3 (p=0.984). This pattern was corroborated in the external test cohort, where model 3 again exhibited significantly better performance than model 1 (p=0.019). Although the performance advantages of model 3 over model 2 (p=0.245) and model 1 over model 2 (p=0.195) did not reach statistical significance, model 3 consistently showed improvements across all evaluated metrics, as detailed in table 3.

### Clinical utility assessment via DCA

**Figure 5:**
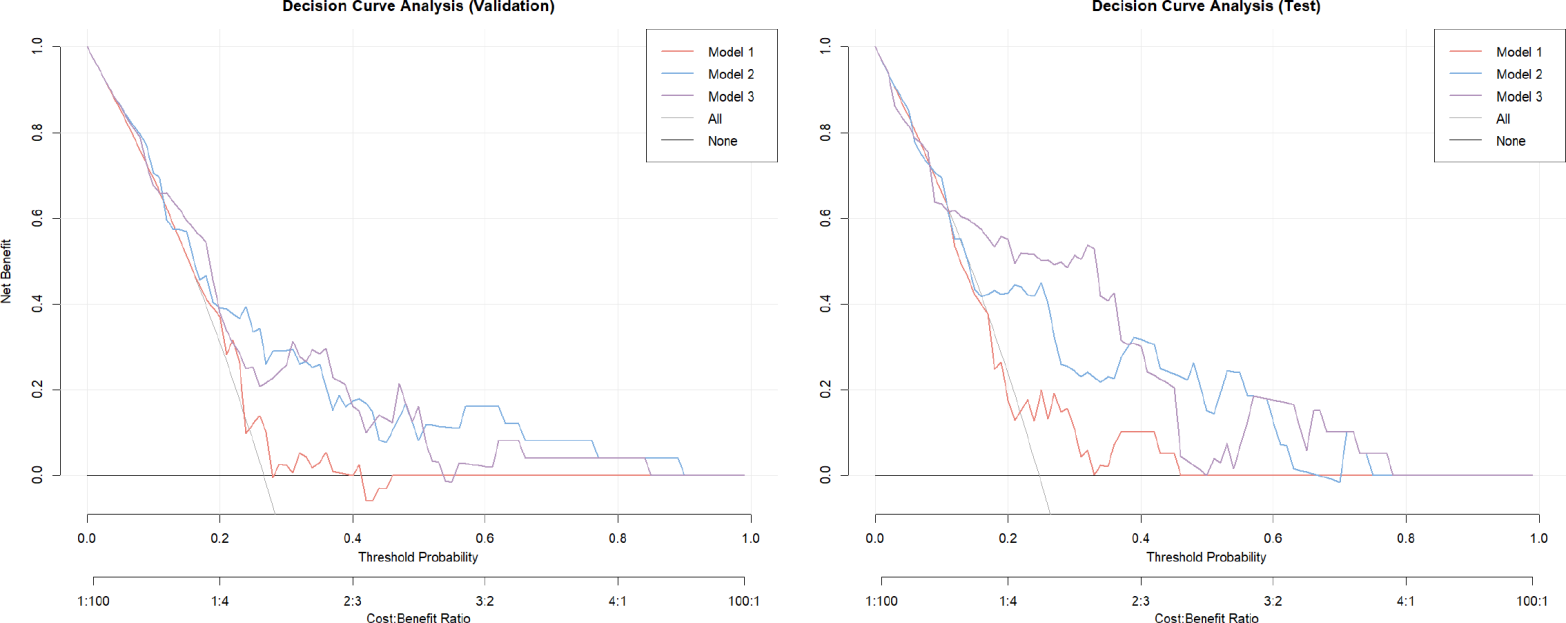
Decision curve analysis for models 1, 2 and 3. Model 1 (clinical factors and LA-EAT volume), model 2 (radiomics model), model 3 (model 1+model 2). Decision curves demonstrating the net benefit of each model for various threshold probabilities. EAT, epicardial adipose tissue; LA, left atrial.

DCA showed that model 3 offered the highest net benefit across various threshold probabilities (0.2–0.4), with positive net benefits extending to thresholds up to 0.5n (figure 5). Although model 2 demonstrated comparable performance, it consistently performed slightly worse than model 3 across the same threshold range. In contrast, model 1 underperformed in all scenarios.

## Discussion

This study aimed to evaluate the association between LA-EAT radiomic features and AF recurrence following AF ablation. Our findings demonstrated that radiomics-based models focusing on LA-EAT outperformed those based on the whole EAT, with the LR model achieving the highest predictive accuracy. LA-EAT volume had a stronger predictive ability for AF recurrence than the total EAT volume.

Clinical predictors included persistent AF, elevated BMI, female sex, and increased BNP/pro-BNP levels. Although elevated BNP/pro-BNP showed no statistically significant association with recurrence risk in univariate analysis, its near-significant effect in multivariable LR (HR=1.09, p=0.052) suggests these biomarkers hold biological relevance as AF recurrence predictors. The observed attenuation in significance could reflect limitations of our dichotomised categorisation, which may obscure subtle dose-dependent relationships evident in continuous analyses. Notably, while advanced age exhibited a paradoxical protective effect (HR=0.99) in our cohort, this finding requires cautious interpretation due to potential confounding factors such as differential treatment intensity across age groups or age-related disparities in controlled comorbidities.^15^ In addition, our study confirms and expands the current knowledge regarding the role of EAT in AF recurrence. Previous studies have shown that clinical factors alone have limited predictive value,^16^ whereas quantitative analysis of EAT yields greater clinical utility.^17^ Specifically, detailed segmentation of LA-EAT has been highlighted as being more predictive of AF recurrence than global EAT quantification.^12 18^ In addition to measuring the EAT volume, enhancement degree and CT dispersion,^19^ radiomics is a promising area for further investigation.

In their pioneering work, Yang *et al* initially established the prognostic significance of LA-EAT for AF recurrence through a single-centre investigation with limited sample size.^13^ Building on this foundation, our study advances the field by implementing an automated, deep learning-based segmentation approach that effectively addresses the inherent limitations of manual delineation while enhancing the reproducibility of radiomics feature extraction. Furthermore, the clinical applicability of our model was rigorously validated through external testing, demonstrating robust generalisability with an AUC of 0.79 in the independent cohort.

Pathophysiologically, EAT alters the structure of the left atrium through mechanisms such as fat infiltration, fibrosis and inflammation, thereby promoting the onset and progression of AF.^20^ These structural changes are synergistic. Studies have demonstrated that the degree of EAT fibrosis (collagen deposition) is significantly and positively correlated with myocardial fibrosis in the LA.^21^ These pathological alterations impair LA function^22^ and destabilise atrial electrical activity, thereby increasing the risk of recurrence. Our study, based on imaging radiomics of LA-EAT, corroborates these findings. Taking the two features with the largest absolute coefficients as examples, the WT.HHH Skewness feature in the LR model had a positive coefficient, indicating that patients with AF recurrence exhibited greater asymmetry in the intensity distribution of LA-EAT. On the other hand, LoG2.3D IMC1 quantifies the dependency and complexity between two probability distributions. The original quantified value was negative, and a larger absolute value reflected greater texture dependency and increased distribution complexity. In our model, the negative coefficient of IMC1 suggested that patients with AF recurrence exhibited greater asymmetry in the intensity distribution of LA-EAT, indicating higher heterogeneity. These radiomic features provide deeper insights into the structural and compositional differences in EAT associated with AF recurrence.

Many cases of AF recurrence after ablation remain undetected owing to the absence of symptoms.^23 24^ However, restoring sinus rhythm can help improve cardiac function and atrial remodelling in affected patients.^25 26^ For individuals identified by the model to have a high probability of recurrence, more proactive monitoring strategies should be considered. For example, implantable cardiac monitoring for high-risk patients can more effectively detect AF episodes following ablation.^27^

This study has limitations. First, structural and functional assessments of LA and LA appendage were not fully available in the current cohort, which might potentially limit the model performance. Future investigation is warranted to explore the benefit of including conventional LA parameters into the prediction model for AF recurrence. Second, we used a non-electrocardiographic-gated PCTV acquisition protocol, which resulted in slight motion artefacts in a small number of slices, potentially affecting the accuracy of EAT quantification. Finally, our study did not employ long-term ECG monitoring, which might have led to the underdetection of asymptomatic AF recurrences.

In conclusion, the radiomic features of LA-EAT can effectively predict the risk of AF recurrence within 1 year of RFA. Furthermore, the LA-EAT radiomics model, when combined with LA-EAT volume and clinical risk factors, showed the highest predictive performance for AF recurrence after ablation therapy.

## Supplementary material

10.1136/openhrt-2025-003364 online supplemental file 1

## Data Availability

Data are available on reasonable request.
