# Supplementary material for: Radiomic features of peri-left atrial epicardial adipose tissue and atrial fibrillation recurrence after ablation
Source: Open Heart. 2025 Jul 8;12(2):e003364. doi: 10.1136/openhrt-2025-003364 (PMC12243630; doi:10.1136/openhrt-2025-003364)
Supplement: online supplemental file 1 [file openhrt-12-2-s001.docx]

**Online supplemental materials**

**Definitions and Diagnostic Criteria for clinic factors**

The International Society of Hypertension (ISH) defines hypertension as systolic blood pressure ≥140 mmHg or diastolic blood pressure ≥90 mmHg in individuals not using antihypertensive medications. The American Diabetes Association (ADA) diagnoses diabetes based on symptoms like polyuria, excessive thirst, unexplained weight loss, or blood glucose levels: random plasma glucose ≥11.1 mmol/L, fasting glucose ≥7.0 mmol/L, or 2-hour glucose ≥11.1 mmol/L. Dyslipidemia is diagnosed per Chinese guidelines with triglycerides >2.3 mmol/L, total cholesterol >6.2 mmol/L, LDL >4.1 mmol/L, or HDL <1.0 mmol/L. WHO defines smoking as regular smoking for ≥6 months, and drinking as daily alcohol intake >60g for men or >40g for women.

Table S1: Automated segmentation performance of LA-EAT with the DL model

|  | Internal Train  (n = 54) | Internal test  (n = 16) | External test  (n = 16) |
| --- | --- | --- | --- |
| DSC | 0.928±0.026 | 0.925±0.025 | 0.910±0.023 |
| HD95 (mm) | 0.650±0.187 | 0.619±0.187 | 0.686±0.092 |

Data are presented as the mean ± standard deviation. EAT, epicardial adipose tissue; DL, deep learning; DSC, dice score coefficient; HD95, Hausdorff distance 95%.

Table S2: Detailed parameter settings for radiomic features extracted by PyRadiomics

| Parameter | Settings |
| --- | --- |
| Image Type |  |
| Original |  |
| LoG | Sigma: [1.0, 2.0, 3.0, 4.0] |
| Wavelet |  |
| Feature Class | Shape; First-order; GlCM; GLRLM; GLSZM; GLDM |
| Setting |  |
| Normalize | true |
| Normalize Scale | 100 |
| Resampling |  |
| Interpolator | 'sitkBSpline' |
| Resampled pixel Spacing: | [1, 1, 1] |
| Image discretization |  |
| Bin width: | 5 |
| First order specific settings |  |
| Voxel array shift | 200 |

Blank means the Pyradiomics default parameters are used. Laplacian of Gaussian filter (LoG); gray-level co-occurrence matrix (GLCM); gray-level size zone matrix (GLSZM); gray-level run length matrix (GLRLM); neighborhood gray-tone difference matrix (NGTDM);gray-level dependence matrix (GLDM)

Table S3: Logistic regression model intercept and coefficients

| Features | Model coefficients |
| --- | --- |
| Logistic regression Intercept | -0.04157 |
| LoG2.3D glcm Imc1 | -0.13933 |
| LoG2.3D glszm SizeZoneNonUniformityNormalized | 0.14564 |
| LoG2.3D glszm SmallAreaLowGrayLevelEmphasis | 0.11753 |
| LoG2.3D gldm HighGrayLevelEmphasis | -0.06323 |
| LoG2.3D firstorder RobustMeanAbsoluteDeviation | -0.02732 |
| LoG2.3D glrlm HighGrayLevelRunEmphasis | -0.05498 |
| LoG3.3D glszm SmallAreaEmphasis | 0.05418 |
| LoG4.3D glszm SmallAreaEmphasis | 0.14354 |
| LoG4.3D firstorder Kurtosis | 0.03598 |
| WT.HLH glcm Autocorrelation | 0.07383 |
| WT.HHH firstorder Skewness | 0.18513 |
| WT.HHH f glrlm LongRunHighGrayLevelEmphasis | 0.02385 |
| WT.LLH gldm SmallDependenceLowGrayLevelEmphasis | -0.01328 |

Laplacian of Gaussian filter (LoG); Wavelet (WT); gray-level co-occurrence matrix (GLCM); gray-level size zone matrix (GLSZM); gray-level run length matrix (GLRLM); neighborhood gray-tone difference matrix (NGTDM);gray-level dependence matrix (GLDM)
